# Defining phenotypic and functional heterogeneity of glioblastoma stem cells by mass cytometry

**DOI:** 10.1172/jci.insight.128456

**Published:** 2021-02-22

**Authors:** Luciano Galdieri, Arijita Jash, Olga Malkova, Diane D. Mao, Patrick DeSouza, Yunli E. Chu, Amber Salter, Jian L. Campian, Kristen M. Naegle, Cameron W. Brennan, Hiroaki Wakimoto, Stephen T. Oh, Albert H. Kim, Milan G. Chheda

**Affiliations:** 1 Department of Medicine,; 2 Center for Human Immunology and Immunotherapy Programs, and; 3 Department of Neurosurgery, Washington University School of Medicine, St. Louis, Missouri, USA.; 4 Biomedical Engineering and Center for Biological Systems Engineering, Washington University in St. Louis, St. Louis, Missouri, USA.; 5 Division of Biostatistics, Washington University School of Medicine, St. Louis, Missouri, USA.; 6 Siteman Cancer Center, Washington University in St. Louis, St. Louis, Missouri, USA.; 7 Department of Neurosurgery, Memorial Sloan Kettering Cancer Center, New York, New York, USA.; 8 Brain Tumor Research Center, Massachusetts General Hospital, Boston, Massachusetts, USA.; 9 Department of Neurology, Washington University School of Medicine, St. Louis, Missouri, USA.

**Keywords:** Oncology, Cancer

## Abstract

Most patients with glioblastoma (GBM) die within 2 years. A major therapeutic goal is to target GBM stem cells (GSCs), a subpopulation of cells that contribute to treatment resistance and recurrence. Since their discovery in 2003, GSCs have been isolated using single-surface markers, such as CD15, CD44, CD133, and α_6_ integrin. It remains unknown how these single-surface marker–defined GSC populations compare with each other in terms of signaling and function and whether expression of different combinations of these markers is associated with different functional capacity. Using mass cytometry and fresh operating room specimens, we found 15 distinct GSC subpopulations in patients, and they differed in their MEK/ERK, WNT, and AKT pathway activation status. Once in culture, some subpopulations were lost and previously undetectable ones materialized. GSCs that highly expressed all 4 surface markers had the greatest self-renewal capacity, WNT inhibitor sensitivity, and in vivo tumorigenicity. This work highlights the potential signaling and phenotypic diversity of GSCs. Larger patient sample sizes and antibody panels are required to confirm these findings.

## Graphical abstract

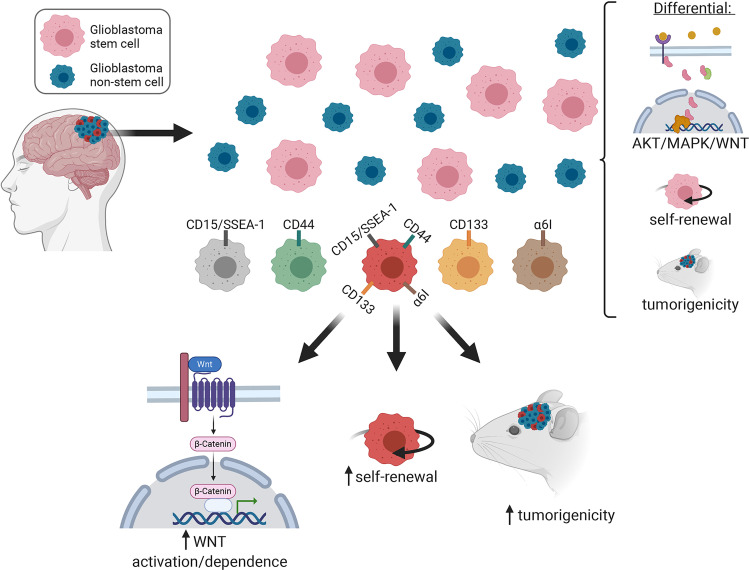

## Introduction

Glioblastoma (GBM) is the most common and aggressive primary brain tumor. Standard therapy includes surgery, radiation, temozolomide chemotherapy, and, more recently, tumor-treating fields (1). Recurrence, on average, occurs 6 months after maximal therapy (2). GBM stem cells (GSCs), also known as tumor-propagating cells or tumor-initiating cells (3), may be one reason for inevitable recurrence, as they are highly resistant to radiation and chemotherapy (4–6). GSCs were first isolated using an antibody against the cell-surface protein CD133 (Prominin-1) (7). CD133^hi^ cells have clonogenic self-renewal capacity and efficiently engraft and form intracranial tumors in immunocompromised mice (8, 9). Although sorting by CD133 enriches for GSC function, CD133^lo^ cells can also exhibit clonogenic self-renewal and asymmetric cell division, albeit less efficiently (10, 11). Alternative single-surface markers such as CD15 (SSEA-1), CD44, α_6_ integrin, and A2B5 may also enrich for the GSC state (12–16). The literature has used the term GSC with varying definitions. We use it here as synonymous with a stem cell marker–bearing GBM cell. GSCs tend to be enriched in serum-free media conditions, often referred to as stem cell media conditions. It remains unknown how GSC populations defined by single-surface markers compare with each other, in terms of intracellular signaling and function and whether expression of different combinations of these markers is associated with differences in the probability of tumor-forming capacity. More broadly, it remains unknown whether all GSCs are alike or have their own hierarchy of function. These issues are important for how we study GBM in vitro and in animal models and understand intratumor and intertumor heterogeneity and treatment resistance.

Mass cytometry is a quantitative analytical technique whereby single cells labeled with antibodies tagged with rare earth metals are ionized and analyzed by time-of-flight mass spectrometry. This largely overcomes the spectral overlap typical of standard flow cytometry, which limits the number of observations possible on a given cell. As such, mass cytometry theoretically enables the use of up to 100 analysis channels, with over 50 currently available heavy metal isotopes to study (17, 18).

We used mass cytometry to evaluate the intracellular states associated with 4 commonly used GSC surface markers, CD15, CD44, CD133, and α_6_ integrin. We measured normal neural stem cell–associated intracellular markers that have also been implicated in GSC proliferation, migration, and tumorigenesis, e.g., Sox2 (19–21), Musashi (22–24), Nanog (25), and Nestin (26) (7, 8, 12). We also probed core developmental pathways that are often activated in GSCs and for which targeted therapies are available, such as PI3K/AKT (27), MEK/ERK (28), JAK/STAT (29), WNT/β-catenin (30, 31), NF-κB (32, 33), and MAPK/P38 (34); their downstream effectors; and cancer-associated markers (Table 1).

To study GBM by mass cytometry, patient samples were quickly dissociated into single cells and fixed prior to analysis to avoid loss of phenotypic markers and cell populations (35, 36). We herein report that GSC subpopulations differ in signaling, self-renewal potential, and in vivo tumorigenicity depending on which surface markers are used to isolate them. We also report that the composition of the overall GSC population shifts in culture, compared with fresh isolates.

## Results

### Mass cytometric analysis of fresh patient samples identified a heterogeneous distribution of GSC subpopulations between patients.

**Figure 1:**
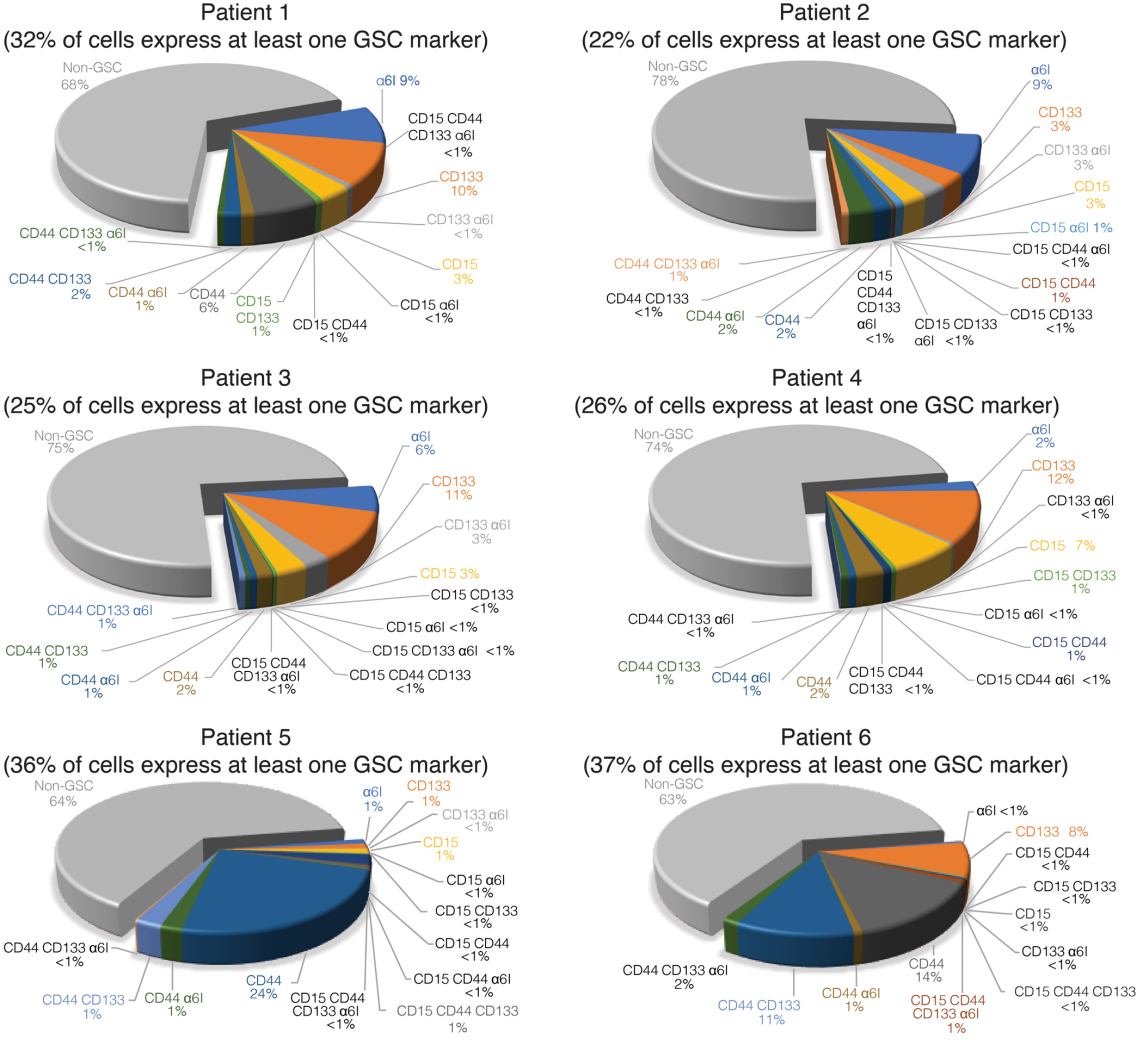
All GSC subpopulations exist in patients. Pie charts demonstrate the percentage of each GSC subpopulation relative to the total number of cells analyzed from each patient sample. The percentage of cells that highly express at least one of CD15, CD44, CD133, or α_6_ integrin is indicated under the patient number. GSC, glioblastoma stem cell.

We obtained fresh tumor samples from the operating room of 6 patients at the time of GBM diagnosis (Table 2). We dissociated tumors into single-cell suspension within 30 minutes after tissue acquisition. We obtained an average of 1.35 × 10^4^ live cells per mg of tissue, and 3 × 10^6^ viable cells were immediately labeled for mass cytometry. To identify GSC subpopulations based on stem cell–surface marker state, using 4 GSC markers and their combinations, we considered 15 theoretical states, assuming each cell can have high or low expression of each marker, and 1 non-GSC state (low expression for all 4 surface markers). For positive and negative controls for cell-surface and intracellular GSC markers, we used the patient-derived GSC line 0308 cultured in Neurobasal media supplemented with growth factors and cultured in DMEM media containing 10% FBS for 6 weeks, respectively. GSCs grown in the presence of FBS are phenotypically distinct from cells grown in serum-free media (37), and FBS-containing media reduced the expression of all 4 surface markers (Supplemental Figure 1; supplemental material available online with this article; https://doi.org/10.1172/jci.insight.128456DS1). From the 6 patient specimens, we identified all 16 possible states (Figure 1). The entire population of GSCs, as defined as high expression of at least one GSC cell-surface marker, comprised an average of 29.6% (range from 22.2% to 37%) of live cells analyzed. We observed a heterogeneous distribution of GSC subpopulations between patients. The range of high expression for each individual marker was 3.3%–9.3% CD15, 3.1%–53% CD44, 6.6%–19% CD133, and 2.0%–16.2% α_6_ integrin. Some populations were rare and represented less than 1% of the entire GSC population, e.g., CD15^hi^CD44^hi^CD133^hi^ and CD15^hi^CD44^hi^CD133^hi^α_6_ integrin^hi^ (Figure 1 and Supplemental Table 1).

**Figure 2:**
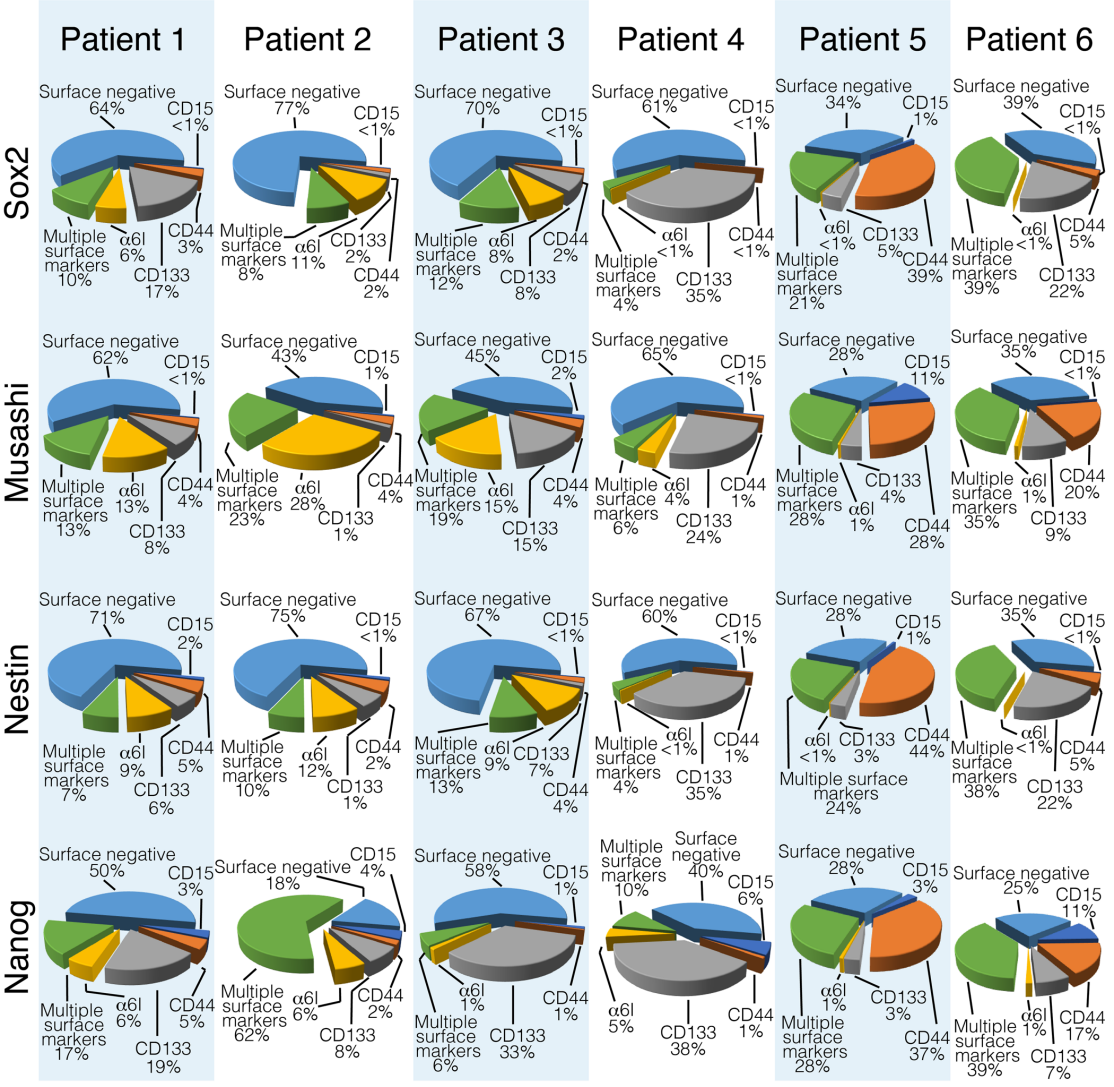
Intracellular neural stem cell–associated proteins are expressed in GSCs and non-GSCs. For each indicated intracellular protein, all cells that highly express it total to 100%. The subpopulation contribution to this total is indicated. GSCs, glioblastoma stem cells.

We also assessed the expression of the intracellular neural stem cell–associated proteins Sox2, Musashi-1, Nestin, and Nanog. We observed that all 4 intracellular markers were expressed in GSCs and non-GSCs (Figure 2). We also found that 14%–50% of the cells expressing any of the 4 neural stem cell–associated intracellular markers also expressed a single GSC cell-surface marker (Figure 2). Conversely, compared with non-GSCs, not all GSC subpopulations had high levels of expression of one of these neural stem cell–associated intracellular markers (Supplemental Figure 2).

### Compared with non-GSCs, fresh GSC subpopulations differed in MEK/ERK, WNT, and AKT pathway activation and had increased WNT and NF-κB activation.

**Figure 3:**
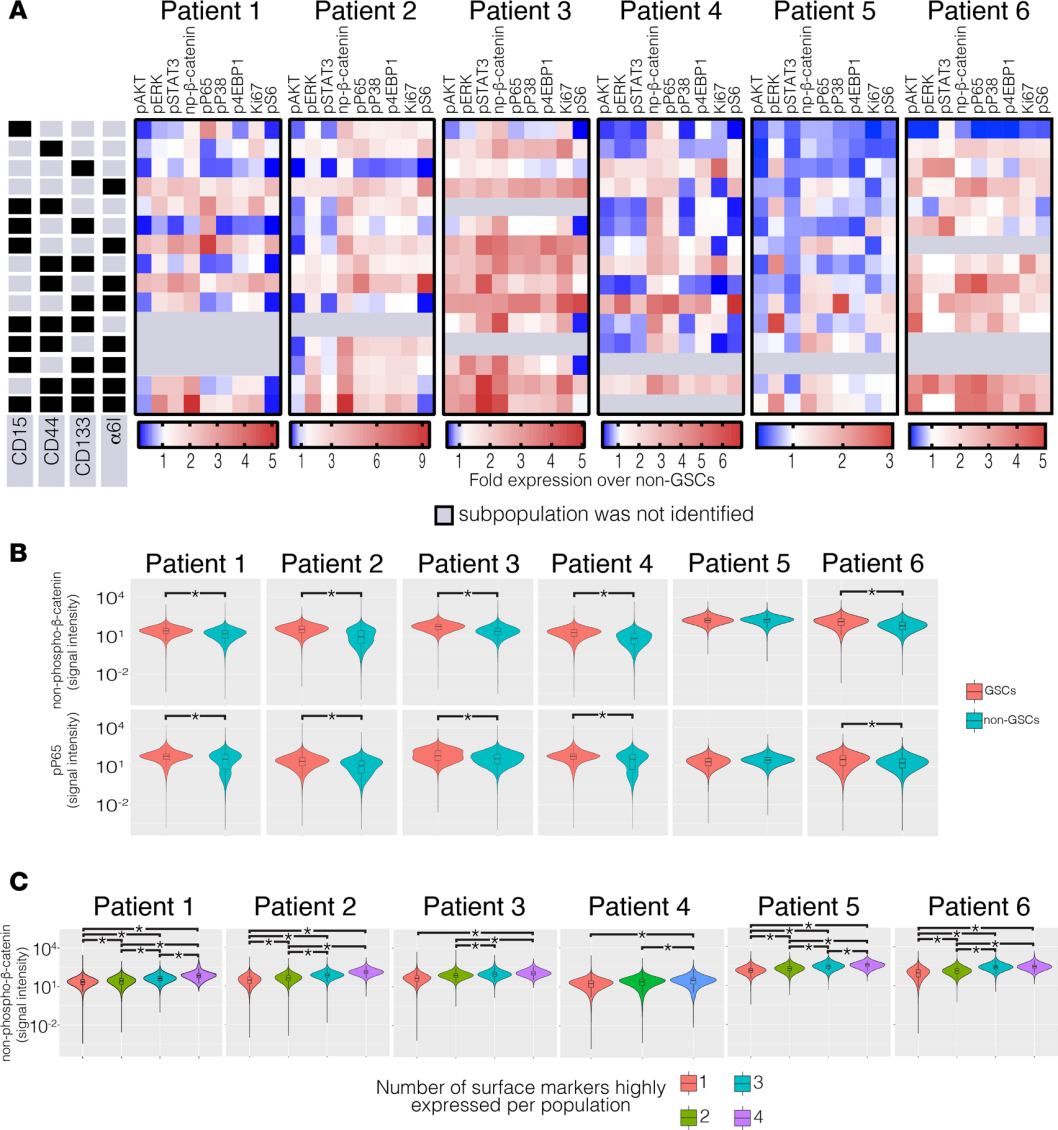
GSC subpopulations have differential activation of MEK/ERK, WNT, AKT, and NF-κB pathways. (**A**) Side panels indicate the expression level (high, black; low, gray) of the cell-surface markers that define each subpopulation. Across each patient, the indicated protein in each subpopulation is shown; heatmaps indicate fold protein expression relative to non-GSCs. Six intracellular pathways (pAKT, pERK, pSTAT3, non–phospho-β-catenin, pP65, and pP38) and three intracellular downstream effectors (Ki-67, p4E-BP1, and pS6) were examined. (**B**) Expression of non–phospho-β-catenin and pP65 in GSCs and non-GSCs, on log scale. Kruskal-Wallis with Mann-Whitney *U* post hoc tests were used, *P < 0.05 vs. non-GSCs. (**C**) Expression of non–phospho-β-catenin in GSCs grouped by the number of highly expressed surface markers, on log scale. Kruskal-Wallis with Bonferroni’s post hoc tests were used; *P < 0.05. GSC, glioblastoma stem cell.

To determine the activation level of intracellular pathways, we used mass cytometry with a panel of 20 antibodies (Table 1). Activation of the PI3K/AKT, MEK/ERK, JAK/STAT, NF-κB, and MAPK/P38 pathways was determined by increased phosphorylation of AKT (pAKT), ERK (pERK), STAT3 (pSTAT3), P65 (pP65), and P38 (pP38), respectively. Activation of the WNT pathway was determined by increased expression of non–phospho-β-catenin. We found that the quadruple-high subpopulation, CD15^hi^CD44^hi^CD133^hi^α_6_ integrin^hi^, had high expression of pERK and non–phospho-β-catenin compared with cells with low expression of surface markers (Figure 3A). In addition, the subpopulation CD44^hi^CD133^hi^α_6_ integrin^hi^ also had consistently high expression of phospho-ERK and non–phospho-β-catenin among all 6 patients. In contrast, CD15^hi^ and CD15^hi^CD133^hi^ subpopulations had consistently low expression of pAKT (Figure 3A).

GSCs as a group had significantly greater WNT activation (*P* < 0.01 patients 1–4 and 6) compared with cells lacking expression of all of the GSC surface markers (quadruple low; Figure 3B). We also tested whether the presence of greater numbers of stem cell–surface markers is associated with greater WNT activation. Combining our patient data and collapsing the subpopulations into single, double, triple, or quadruple-high states from each patient sample, and correcting for multiple hypothesis testing, we found that increased numbers of surface markers were associated with increased expression of non–phospho-β-catenin (Figure 3C; *P* values in Supplemental Table 2), a transcription factor that is activated when a Wnt ligand binds to the Frizzled and LRP6 coreceptors (38). The quadruple-high subpopulation, CD15^hi^ CD44^hi^ CD133^hi^ α_6_ integrin^hi^, had the highest protein expression of non–phospho-β-catenin in samples from patients 1, 2, 3, 5, and 6. In patient 4, which lacked the quadruple-high subpopulation, the subpopulations with high expression of any 3 surface markers had the greatest abundance of non–phospho-β-catenin. Additionally, GSCs as a group had increased expression of pP65 compared with non-GSCs, a surrogate of NF-κB pathway activation (33) (Figure 3B; *P* < 0.01 patients 1–4 and 6). Myeloid cells in the tumor microenvironment did not likely skew our interpretation (Supplemental Figure 3).

### Short term culture was associated with both loss and gain of GSC subpopulations.

**Figure 4:**
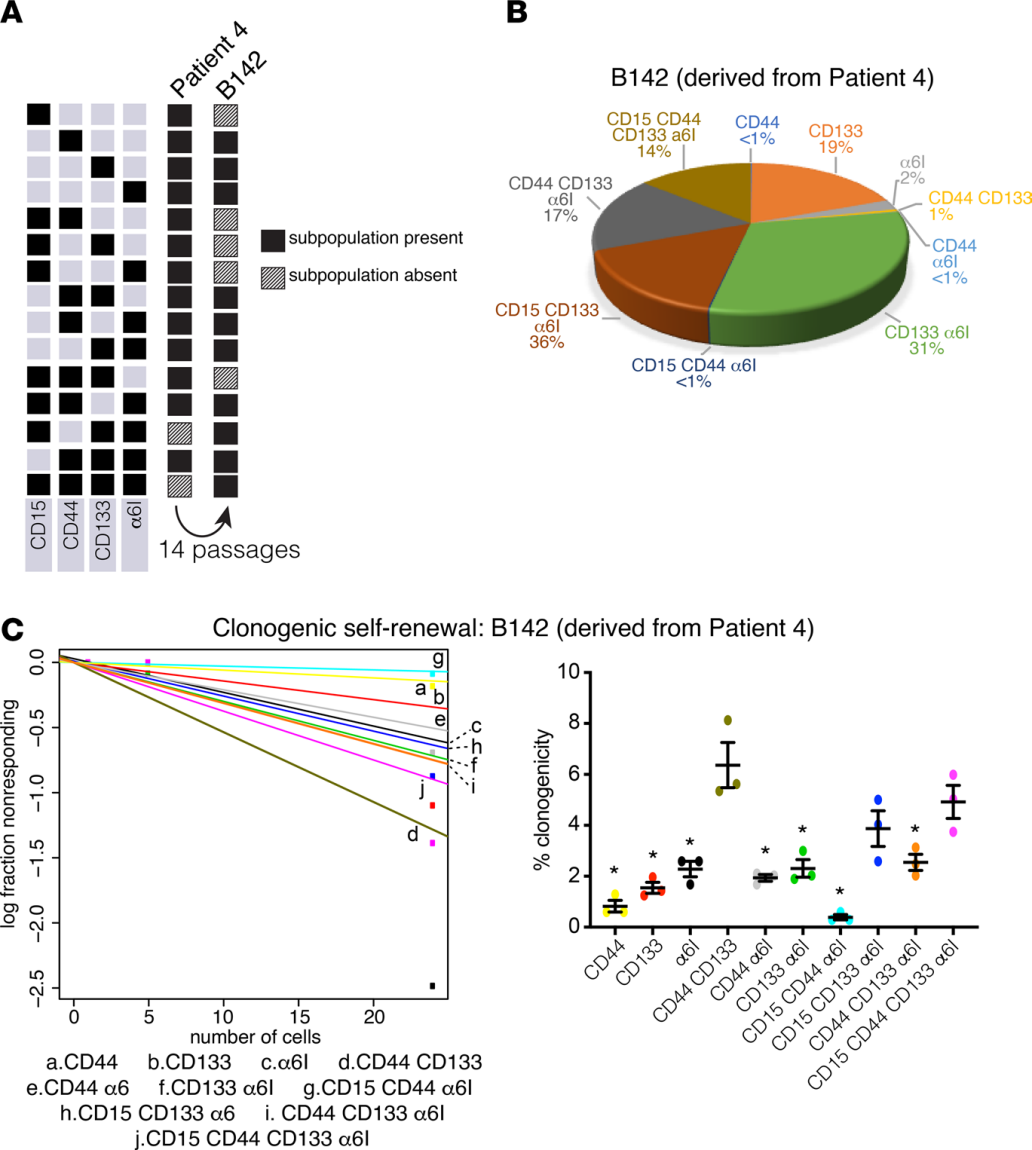
GSC populations are lost and gained in culture, and CD15^hi^CD44^hi^CD133^hi^ α_6_ integrin^hi^ (quadruple high) cells and CD44^hi^CD133^hi^ cells derived from patient 4 are the most clonogenic. (**A**) B142 GSCs were derived from patient 4. Black indicates the presence of the indicated GSC subpopulation; hash pattern indicates its absence. (**B**) Pie chart indicates the percentage of each GSC subpopulation relative to the total B142 population. (**C**) Clonogenic self-renewal for B142 cell line was assessed by extreme limiting dilution analysis (24, 5, and 1 cells per well; 12–18 replicates per dilution). The experiment was repeated 3 times, and the results are shown as mean ± SEM. ANOVA with Tukey’s post hoc tests were used to assess the significance of differences between each GSC subpopulation. *P < 0.05 vs. quadruple-high. GSC, glioblastoma stem cell.

We were only able to derive one GSC line from our 6 patient specimens (patient 4, GSC line B142). We test whether GSC subpopulation compositions were perturbed by culture conditions. Using FACS (Supplemental Figure 4), we observed that although the initial specimen contained 14 GSC states, after short-term culture (14 passages), only 10 subpopulations were detected (Figure 4A). Interestingly, although we failed to detect 5 GSC subpopulations that had existed in the fresh sample, 2 subpopulations were detectable in the cultured sample (Figure 4, A and B).

### GSC subpopulations in short-term and long-term culture had different self-renewal capacities, depending on the cell-surface markers used to define them.

Using B142, we measured the relative rates of clonogenic self-renewal of each sorted GSC population using the extreme limiting dilution assay (ELDA) (39, 40). Clonogenic potential ranged from 0.4% to 6.3% (Figure 4C). The cells expressing high levels of CD44 and CD133 only (CD44^hi^CD133^hi^) and all 4 markers (CD15^hi^CD44^hi^CD133^hi^α_6_ integrin^hi^) had the greatest degree of self-renewal capacity, with clonogenic potential of 6.3% and 4.9%, respectively (Figure 4C; CD44^hi^, *P* < 0.01; CD133^hi^, *P* < 0.01; α_6_ integrin^hi^*, P* = 0.0179; CD44^hi^α_6_ integrin^hi^, *P* < 0.01; CD133^hi^α_6_ integrin^hi^, *P* = 0.0194; CD15^hi^CD44^hi^α_6_ integrin^hi^, *P* < 0.01; CD44^hi^CD133^hi^α_6_ integrin^hi^, *P* = 0.0417).

**Figure 5:**
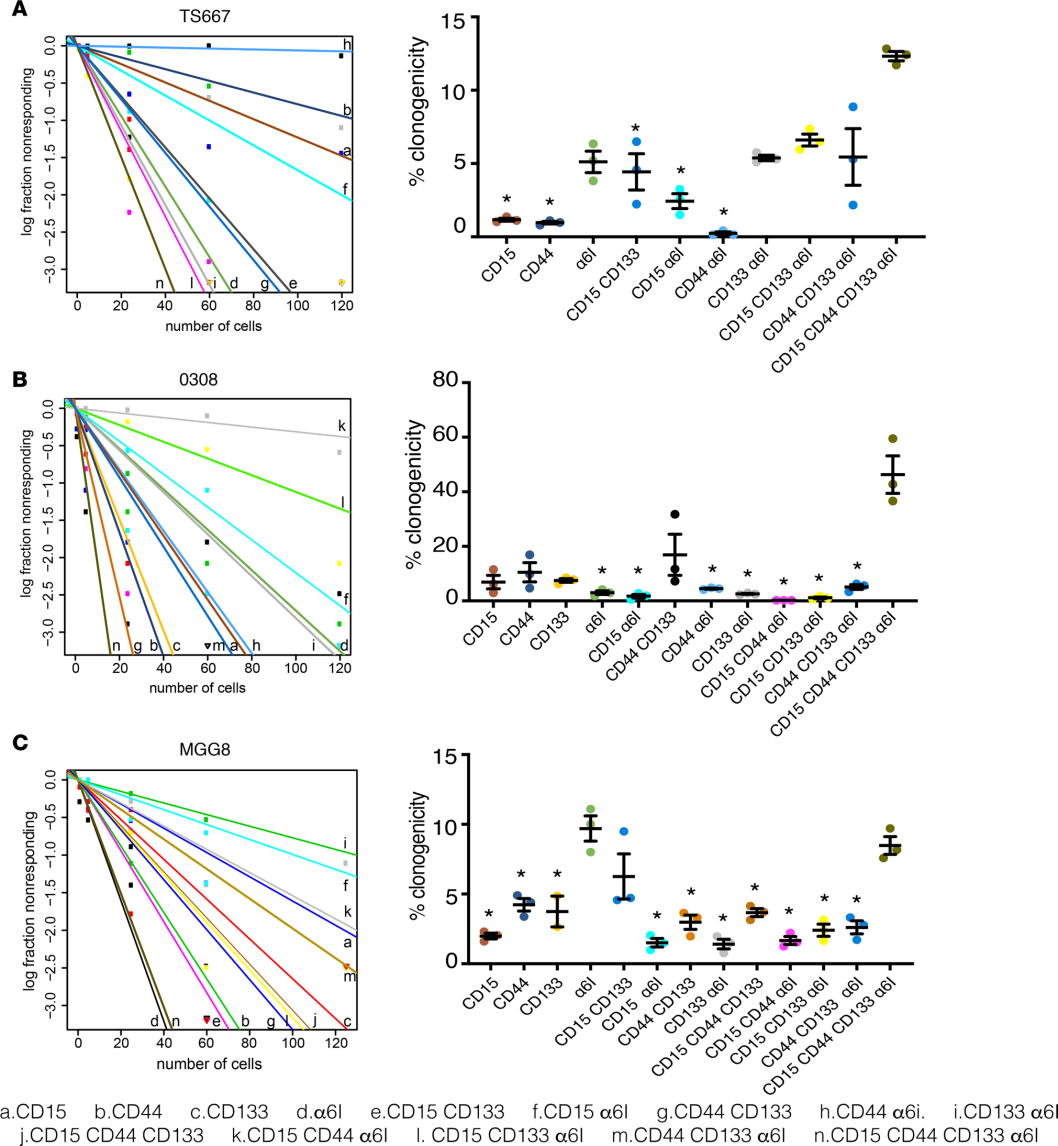
GSC subpopulations vary in their self-renewal potential. Frequency of clonogenic cells was assessed by extreme limiting dilution analysis using GSC subpopulations derived from (**A**) TS667, (**B**) 0308, and (**C**) MGG8 lines (120, 60, 24, 5, and 1 cells per well; 12–18 replicates per dilution). The experiment was repeated 3 times, and results are shown as mean ± SEM. ANOVA with Tukey’s post hoc tests were used to assess the significance of differences between each GSC subpopulation; *P < 0.05 vs. quadruple-high. GSC, glioblastoma stem cell.

Similarly, we identified from 3 patient-derived GSC lines in long-term culture (Table 3) 13 of the 16 possible states (Supplemental Figure 5). Clonogenic potential as measured by ELDA ranged from 0.3% to 12.3% in TS667 GSCs (Figure 5A); 0.3% to 46.3% in 0308 GSCs (Figure 5B); and 1.4 % to 9.7% in MGG8 GSCs (Figure 5C). For TS667 and 0308, the quadruple-high subpopulation had the greatest degree of in vitro self-renewal capacity (Figure 5, A and B) (TS667, CD15^hi^, *P* < 0.01; CD44^hi^, *P* < 0.01; CD15^hi^CD133^hi^, *P* = 0.0437; CD15^hi^α_6_ integrin^hi^, *P* = 0.0104; CD44^hi^α_6_ integrin^hi^, *P* < 0.01; 0308, CD15^hi^α_6_ integrin^hi^, *P* < 0.01; CD44^hi^α_6_ integrin^hi^, *P* < 0.01; CD133^hi^α_6_ integrin^hi^, *P* < 0.01; CD15^hi^CD44^hi^α_6_ integrin^hi^, *P* < 0.01; CD15^hi^CD133^hi^α_6_ integrin^hi^, *P* < 0.01). For MGG8, both the α_6_ integrin high and the quadruple-high subpopulations had the greatest extent of clonogenic potential (Figure 5C) (CD15^hi^, *P* < 0.01; CD44^hi^, *P* < 0.01; CD133^hi^, *P* < 0.01; CD15^hi^α_6_ integrin^hi^, *P* < 0.01; CD44^hi^CD133^hi^, *P* < 0.01; CD133^hi^α_6_ integrin^hi^, *P* < 0.01; CD15^hi^CD44^hi^CD133^hi^, *P* < 0.01; CD15^hi^CD44^hi^α_6_ integrin^hi^, *P* < 0.01; CD15^hi^CD133^hi^α_6_ integrin^hi^, *P* < 0.01; CD44^hi^CD133^hi^α_6_ integrin^hi^, *P* < 0.01).

### GSC subpopulations differed in intracellular pathway activation states and downstream effectors in vitro, depending on the cell-surface markers used to define them.

**Figure 6:**
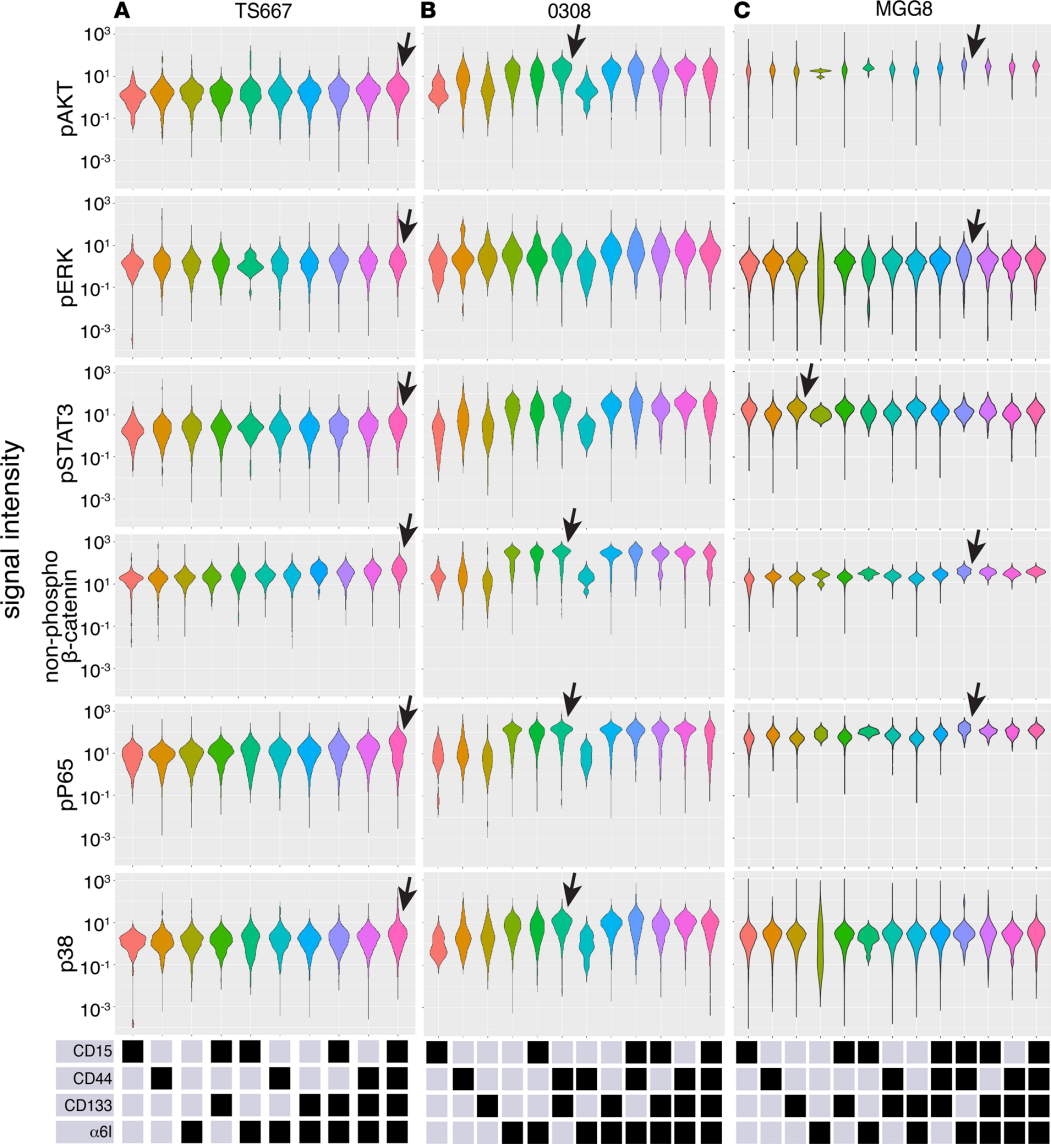
Mass cytometry detects core signaling within single cells among 13 GSC subpopulations from 3 patient-derived lines in long-term culture. Violin plots indicate protein levels for 6 intracellular pathways (pAKT, pERK, pSTAT3, non–phospho-β-catenin, pP65, and pP38) in (**A**) TS667, (**B**) 0308, and (**C**) MGG8 cells. Bottom panels show the levels (high, black; low, gray) of the cell-surface markers defining each subpopulation. Arrows highlight the subpopulation with the highest average protein abundance, as discussed in the text. GSC, glioblastoma stem cell.

For mass cytometry studies of GSC subpopulations, we used antibodies against the same 4 cell-surface markers above. We also assessed signal activation using antibodies against pAKT, pERK, pSTAT3, non–phospho-β-catenin, pP65 (NF-κB), and pP38. In TS667 GSCs, the quadruple-high subpopulation, CD15^hi^CD44^hi^CD133^hi^α_6_integrin^hi^, had the greatest average activation of all 6 pathways studied (Figure 6A). In particular, similar to the fresh operating room GSC specimens, this subpopulation of TS667 had the highest abundance of pERK and non–phospho-β-catenin compared with other GSC subpopulations (Figure 6A). In 0308 GSCs, the CD44^hi^CD133^hi^ subpopulation presented the strongest activation of the PI3K/AKT, WNT/β-catenin, NF-kB, and MAPK/P38 pathways (Figure 6B). In MGG8 GSCs, the CD15^hi^CD44^hi^α_6_ integrin^hi^ subpopulation had the strongest activation of the PI3K/AKT, WNT/β-catenin, and NF-kB pathways (Figure 6C).

**Figure 7:**
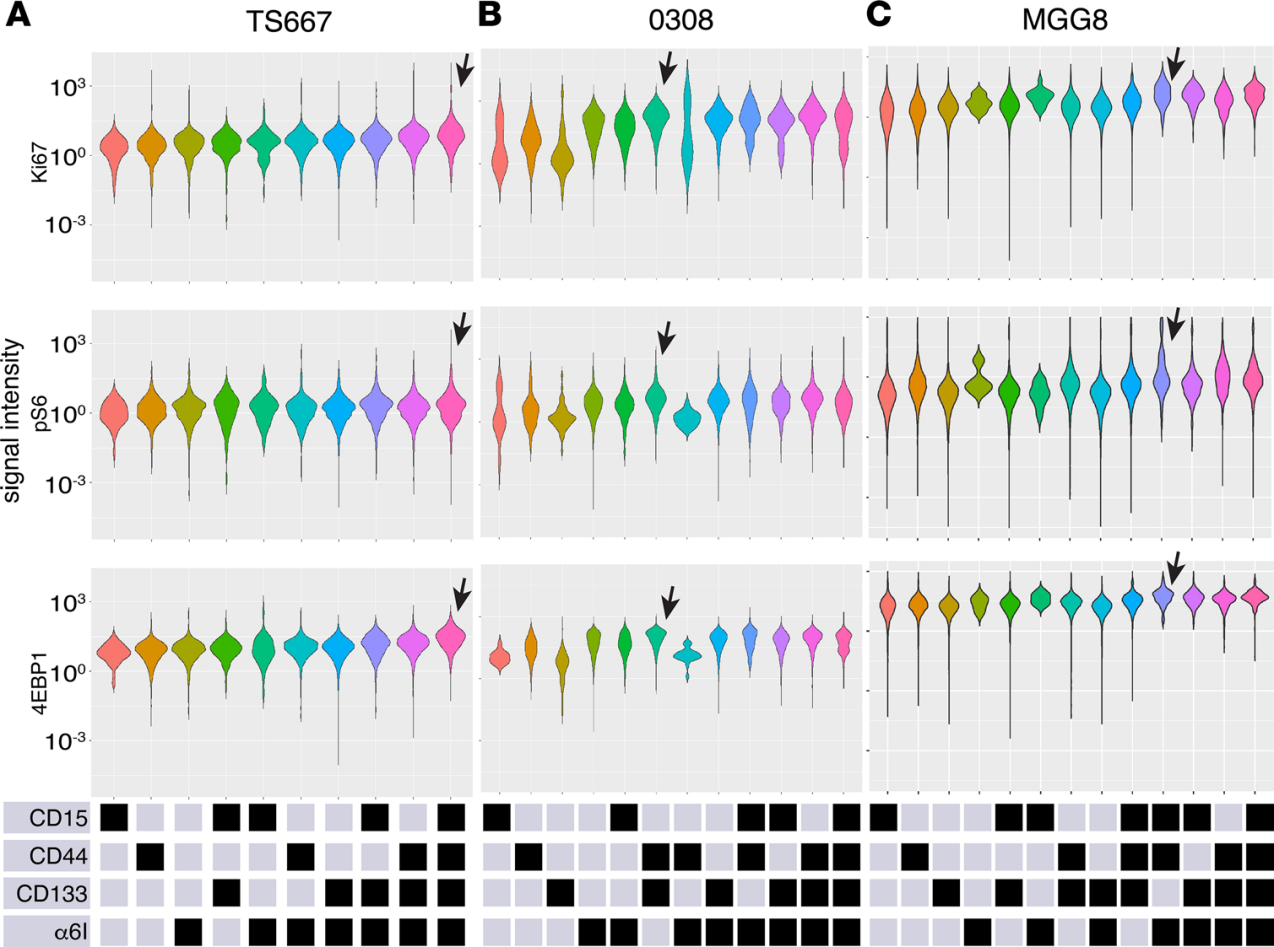
GSC subpopulations with increased activation of PI3K/AKT, WNT/β-catenin, NF-κB, and MAPK/P38 core signaling pathways have increased expression of markers for cell proliferation and translation. Violin plots indicate protein status of markers of cell proliferation (Ki-67) and translation (p4E-BP1, pS6) by GSC subpopulation in (**A**) TS667, (**B**) 0308, and (**C**) MGG8 cells. Bottom panels show the levels (high, black; low, gray) of the cell-surface markers defining each subpopulation. Arrows highlight the subpopulation with the highest average protein expression, as discussed in the text. GSC, glioblastoma stem cell.

To determine whether GSC subpopulations may differ in cell biological processes, we assayed markers of cell proliferation (Ki-67) (41–43) and RNA translation (p4E-BP1, pS6) (44, 45). In TS667 GSCs, expression of Ki-67, pS6 and p4E-BP1 were the highest in the quadruple-high subpopulation (Figure 7A). In 0308 GSCs, the CD44^hi^CD133^hi^ subpopulation had high expression of Ki-67, pS6, and p4E-BP1 (Figure 7B). In MGG8 GSCs, the triple-high, CD15^hi^CD44^hi^α_6_ integrin ^hi^, and the quadruple-high, CD15^hi^CD44^hi^CD133^hi^α_6_ integrin^hi^, subpopulations had greatest expression of Ki-67, pS6, and p4E-BP1 (Figure 7C). In summary, in standard culture conditions, high expression of a single-cell surface marker was inadequate to identify the state with greatest self-renewal capacity or greatest intracellular pathways activation.

**Figure 8:**
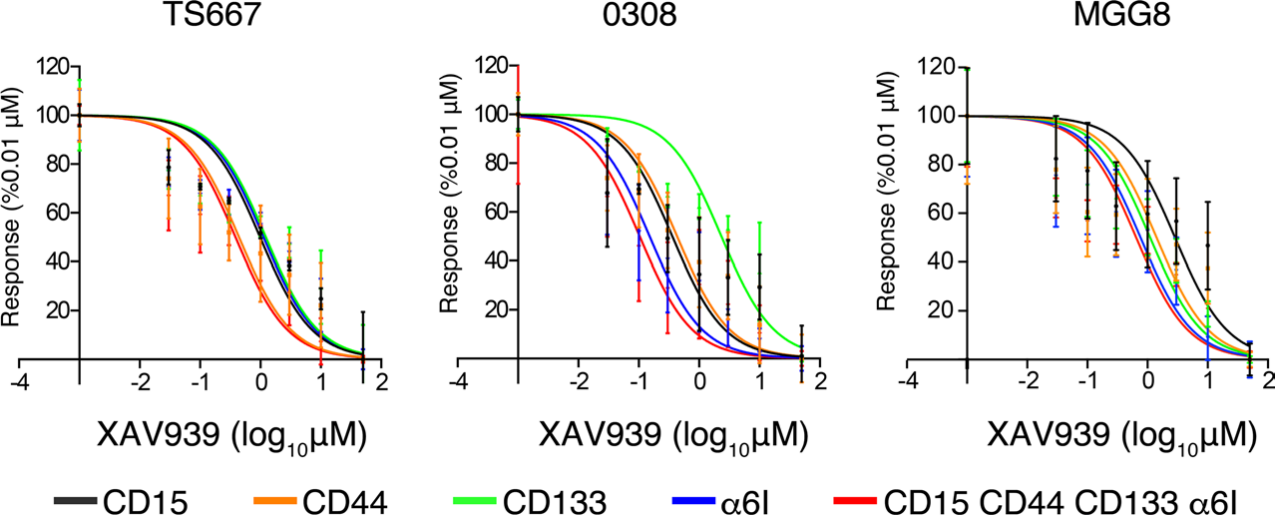
Quadruple-high GSCs are sensitive to WNT inhibition. TS667, 0308, and MGG8 GSC subpopulations were incubated for 5 days with the canonical WNT pathway inhibitor XAV939 and cell viability was measured. The experiment was repeated 3 times, and results are shown as means ± SEM. GSCs, glioblastoma stem cells.

Given that we observe heterogenous activation of WNT signaling in patient samples and cell lines, we next investigate whether GSC subpopulations have differential sensitivity to WNT inhibition. We treated cells with the canonical WNT inhibitor XAV939, which increases degradation of β-catenin and decreases β-catenin–mediated transcription (46). We found that the quadruple-high cells were more sensitive to WNT inhibition than α_6_ integrin^hi^ cells in TS667 (CD15^hi^, CD44^hi^, and CD133^hi^, nonsignificant; α_6_ integrin^hi^, *P* = 0.042), CD15^hi^, CD44^hi^, and CD133^hi^ cells in 0308 (CD15^hi^, CD44^hi^, and CD133^hi^, *P* < 0.01; α_6_ integrin^hi^, nonsignificant). We found no significant sensitivity to WNT inhibition in MGG8 GSC subpopulations (Figure 8).

### GSC subpopulations differed in their in vivo tumorigenicity.

**Figure 9:**
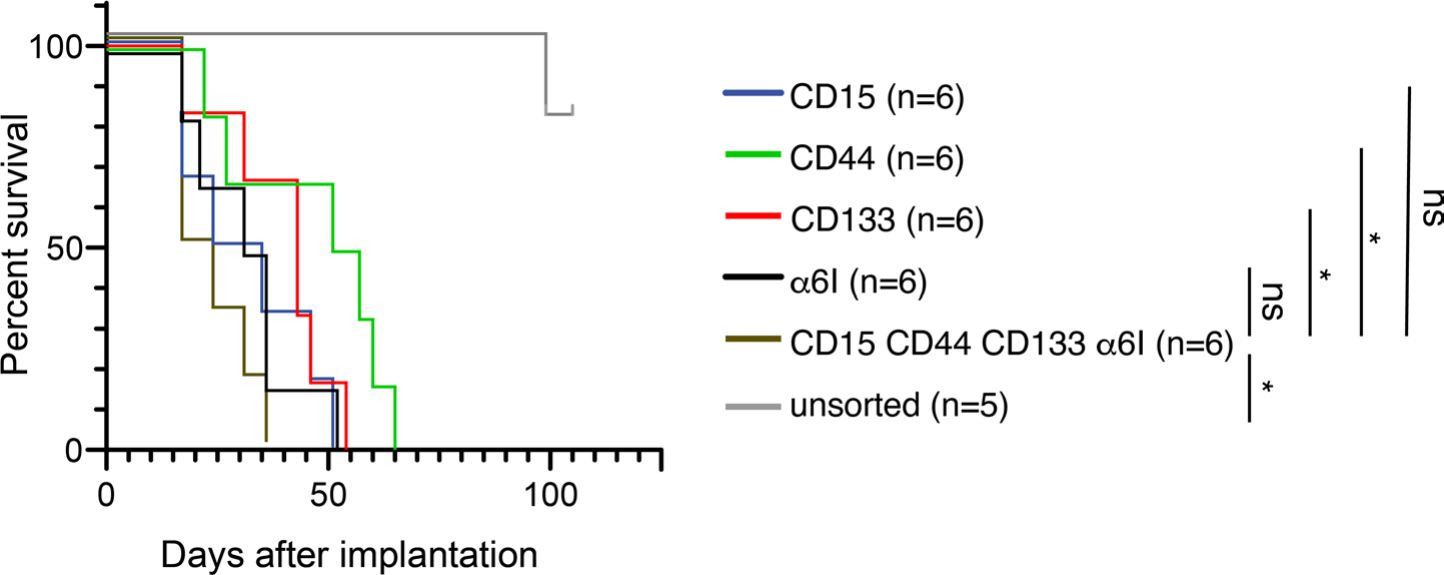
The quadruple-high subpopulation has increased in vivo tumorigenicity. 500 cells of each indicated GSC subpopulation of MGG8 were implanted in NCG mice (*n* = 6). Log-rank test was used to assess the significance of differences between each GSC subpopulation; *P < 0.05 vs quadruple-high cells. GSC, glioblastoma stem cell.

We used a murine intracranial implantation assay to examine whether distinct GSC-associated cell-surface marker profiles are associated with differences in in vivo tumorigenesis. Using the MGG8 patient GSC line, we used magnetic beads and FACS to enrich and isolate subpopulations based on single-surface markers or high expression of all 4 markers, and compared them with unsorted cells grown in standard GSC-enriching media conditions. Upon implantation into the right frontal lobes of NCG female immunodeficient mice (NOD-*Prkdc^em26Cd52^Il2rg^em26Cd22^*/NjuCrl), we followed mice for survival. The quadruple-high subpopulation had the shortest median survival (20.5 days) compared with unsorted (median beyond 100 days, *P* < 0.01). The cells expressing single markers were also more aggressive than the unsorted cells: α_6_ integrin (29.5 days, *P* < 0.01), CD15 (33.5 days, *P* < 0.01), CD133 (43 days, *P* < 0.01), or CD44 (53 days, *P* = 0.0802) (Figure 9). Mice implanted with CD133^hi^ (*P* = 0.0183) or CD44^hi^ (*P* = 0.0209) cells had significantly longer survival than quadruple high as well. Together, these data suggest that even when cells are grown in stem cell–promoting media conditions, upon implantation these unsorted cells had different growth dynamics in vivo than surface marker–enriched cells. Additionally, there may be important in vivo differences between quadruple-high cells and specific subpopulations.

## Discussion

### The CD15^hi^CD44^hi^CD133^hi^ α_6_ integrin^hi^ subpopulation was enriched for GSC characteristics.

We used mass cytometry to characterize the single-cell protein signaling status of fresh GSCs. This may prove a valuable addition to single-cell RNA sequencing in understanding GBM biology and heterogeneity. Single-cell RNA sequencing can detect rare GSC populations cells and transcriptional activation of pathways (47); however, it does not render a clear observation of proteomic intracellular signaling. A multiomic approach can better clarify GBM biology and heterogeneity.

Because GSCs may be one reason for inevitable recurrence in GBM, single-cell analysis of protein states in heterogeneous GSCs may lead to GSC subpopulation–specific therapies. Bulk proteomic analysis using mass spectrometry with patient-derived GSCs can identify differential expression of proteins and phosphoproteins. Recent mass spectrometry studies found increased protein phosphorylation, including the histone methyltransferase enhancer of zeste homolog 2 and the cell motility protein hyaluronan-mediated motility receptor in GSCs compared with neural stem cells from the adult human brain (48); TGF-β receptor type 2 in GSCs grown with EGF compared with GSCs grown in the presence of serum (49); and activation of S6K pathways in GBM cells compared with non-GSCs (50). Proteomics studies also associated the single amino acid variants S1559T in phosphatidylinositol-3,4,5-trisphosphate dependent Rac exchange factor 1 and V632A in dynein axonemal assembly factor 5 with increased risk of GBM (51). However, these studies were done in bulk cells and did not allow single-cell resolution to identify GSC subpopulations and analyze their proteome. In its ability to enable single-cell analysis of the signaling status of proteins, mass cytometry adds granular context to bulk transcriptional and proteomic analysis.

Individual or double positive expression of cell-surface markers has been widely studied (9, 14, 16, 52), but multidimensional stem cell–surface marker studies in GBM are rare and have only been performed in vitro (53). By using 4 stem cell–surface markers, we found 15 states of GSCs exist, each with different levels of activation of core signaling pathways in both patient samples and cell lines. We found that the quadruple-high subpopulation, CD15^hi^CD44^hi^CD133^hi^α_6_ integrin^hi^, had the highest capacity for clonogenic self-renewal in 2 of 4 GSC lines in culture (Figures 4 and 5). α_6_ integrin ^hi^ and CD44^hi^CD133^hi^ also had high clonogenic capacity, but other subpopulations did not follow a clear pattern of surface marker combination and clonogenic potential.

The quadruple-high cells exhibited the highest activation of MEK and WNT pathways among GSC subpopulations in patient samples 1, 2, and 3 and patient samples 1, 2, 3, 5, and 6, respectively, and in the long-term cultured TS667 GSCs. To put this in context, it is known that GSC sphere formation requires ERK activation (28), and GSC tumorigenic capacity and self-renewal requires both the WNT activation and the crosstalk between MEK/ERK and PI3K/AKT (28, 54). EGFR is commonly amplified or mutated in GBM (28) and MEK/ERK and PI3K/AKT are downstream of EGF signaling (55). Activation of MEK/ERK and PI3K pathways suppresses apoptosis (56) and cellular differentiation (28) while promoting cellular proliferation (57). In addition, depletion of the Wnt secretion protein Evi/Gpr177 in both glioma and GSCs decreases cell proliferation and apoptosis (54). Taken together, increased MEK/ERK and WNT activation in the quadruple-high subpopulation suggests that inhibiting these pathways may be clinically useful in targeting this highly clonogenic subset of glioma cells.

In vivo, the quadruple-high GSCs were the most aggressive, along with the α_6_ integrin^hi^. Our results do not support a clear linear relationship between number of surface markers present and tumorigenicity. For example, CD15^hi^α_6_ integrin^hi^ was not particularly more clonogenic than CD15^hi^CD133^hi^α_6_ integrin^hi^ or CD15^hi^ was not more clonogenic than CD15^hi^CD133^hi^. GSCs may represent a plastic state that can be adopted by cancer cells in response to environmental cues rather than a clonal entity defined by stable surface markers expression and distinct phenotypes (58, 59). It is important to consider the possible effects of media conditions, secreted factors, and the tumor microenvironment on this plasticity. Another point worth noting is that our experimental design did not put “unsorted” cells through the process of flow cytometry, and we cannot rule out that the intervention of flow cytometry did not enhance in vivo tumorigenicity of sorted cells.

### From the 6 pathways studied, WNT/β-catenin and NF-κB were the main pathways associated with GSC identity.

All GSC subpopulations from fresh tumor samples had more activation of WNT/β-catenin signaling (indicated by non–phospho-β-catenin) than non-GSC components of the tumor, suggesting that activation of this pathway may be a distinct feature of GSCs. β-Catenin–mediated transcriptional activity is required for self-renewal frequency through interaction with the transcription factor TCF7L2 and disruption of this interaction reduces tumor volume of subcutaneous GSC xenografts (60). Among GSCs, the quadruple high, CD15^hi^CD44^hi^CD133^hi^α_6_ integrin^hi^ cells, had the greatest activation (Figure 3A), which may explain their increased self-renewal capacity in vitro and increased in vivo tumorigenic capacity. These data corroborate that in previous work that demonstrated that accumulation of active non–phospho-β-catenin due to WNT stimulation contributes to differentiation arrest and maintenance of the self-renewal capacity in mouse neural stem cells and malignant glioma patient samples (31). Recent findings suggest that instead of a subpopulation hierarchy, GSCs are capable of transiting between GSC states (58). Although there might not be a unipotent and irreversible subpopulation, the increased clonogenicity together with increased in vitro and ex vivo WNT activation in the quadruple-high GSC suggest that the degree of plasticity might be associated with WNT signaling and tumorigenic potential.

In vivo limiting dilution tumor formation assays have demonstrated that CD133-positive tumor cells are highly tumorigenic in brains of immunocompromised mice, whereas CD133-negative cells seldom form detectable tumors (7, 61). However, our work and previous results (10–15) suggest that not all CD133-containing populations have increased clonogenicity. In fact, our work, although not testing in vivo limiting dilutions, suggests that quadruple-high GSCs had the highest clonogenic renewal. This is consistent with the finding that decreased activation of WNT/β-catenin pathway inhibits proliferation and GBM sphere formation (62).

Our findings also reveal NF-κB activation in GSCs in vivo. We observed increased phosphorylation of the NF-κB subunit, P65, in GSCs from all 6 patients, compared with cells devoid of the 4 surface markers. NF-κB is activated in many human tumors, including glioma (63). In GSCs, the phosphorylation of P65 is increased due to overexpression of the A20 protein (TNFAIP3), a mediator of the NF-κB pathway and cell survival (64), and GSCs in culture have increased phosphorylation and nuclear localization of P65, with resultant increased expression of NF-κB–regulated genes (65) and associated therapeutic resistance (33). Inhibition of P65 phosphorylation in combination with TMZ increases GBM cell apoptosis in vitro compared with TMZ alone (66). The increased P65 phosphorylation we found in GSCs suggests that NF-κB can be used as a potential target to increase TMZ sensitivity of the treatment-resistant GSCs.

We expected to find increased activation of PI3K/AKT, MEK/ERK, JAK/STAT, and MAPK/P38 pathways in cell with increased clonogenic potential. However, we found no distinguishable difference in expression of pAKT, pERK, pSTAT3, and pP38 between the GSC subpopulations among the fresh patient specimens we studied. Our expectations were based on studies using longer term cultures of GSCs in which AKT drives renewal in GSCs in vitro (67). Similarly, JAK/Stat pathway activation is required for in vitro proliferation and self-renewal of patient-derived GSCs (68), whereas Stat3 inhibition decreases expression of neural stem cell transcription factor, Olig2, and inhibits neurosphere formation in GSCs (68). We also expected patient GSCs would have P38 inactivation because inhibition of P38 signaling maintains stemness of patient-derived CD133-positive cells (34). Instead, most GSC subpopulations from patients 2, 3, and 6 showed increased phosphorylated P38. The absence of differences in these pathways in fresh patient specimens was at odds with what we observed in our 2 long-term cultures. Larger numbers of fresh specimens will add more clarity to these observations; however, these findings may highlight the differences between cells in situ and in culture.

### Intracellular neural stem cell–associated proteins were expressed in GSC and non-GSC cells.

Sox2, Musashi-1, Nestin, and Nanog have been considered intracellular markers of the GSC state because of their high expression in neurosphere cultures and previous reports that they are required for maintenance of GSC identity (8, 20, 25, 69). In contrast, we found that the 4 intracellular markers were expressed in cells with and without surface markers associated with the GSC state (Figure 2). Additionally, compared with their quadruple-negative counterparts, not all GSC subpopulations had high levels of expression of all stem cell–associated intracellular markers (Supplemental Figure 2), suggesting that high levels of these intracellular markers are not necessarily linked to the surface marker–defined GSC state in vivo and regulate genes expression and signaling involved in GBM malignancy in both non-GSCs and GSCs (70). Together, although our study includes a small number of patient samples, it does not support the one-to-one correspondence of high intracellular expression of neural stem cell proteins with cell-surface expression of GSC markers. However, it is possible that there exists an expression threshold of intracellular neural stem cell expression that aligns more appropriately with surface marker–defined states.

### Mass cytometry used to study GSC biology.

This work demonstrates the utility of mass cytometry to characterize GSC signaling at the single-cell level in fresh specimens and longer term cultures. A point worth noting is that GSCs derived from patient 4 and placed in short-term culture differed substantially from the GSCs present at diagnosis, in terms of signaling and enrichment of cell states with high expression of CD133. Although this is but one example, these observations demonstrate that GSC identity may drift while in culture; this corroborates a bulk RNA-Seq study demonstrating GSCs in culture develop distinct gene expression and epigenetic profiles from their parental tumors (32). These differences may represent the selective pressures of standard media, particularly with its high concentrations of growth factors, glucose, and glutamine. It is worth considering this as we develop GSC-targeting therapies based largely on work in tissue culture or using cells from culture engrafted into mice. Our observation that several GSC subpopulations were present in culture that were not observed initially ex vivo may mean they either were present initially but below limits of detection or reflect that the GSC states, as defined by surface markers, are fluid.

Larger antibody panels and greater sample sizes will provide a clearer understanding of GSC heterogeneity. For example, GSC subpopulations may vary at the single-cell level, in their degree of expression of commonly amplified or mutated receptors, such as EGFR and PDGFRα. Understanding oncoprotein expression at the single-cell level will inform our interpretation of the failures of targeted therapies in patients with brain tumors. Additionally, including antibodies specific to oncoproteins, such as EGFRvIII or IDH1^R132H^, will assist in differentiating tumor cells compared with nontransformed cells in the microenvironment. Finally, we expect that broader mass cytometry antibody panels will identify heterogeneous expression of intracellular stem cell and precursor marker expression beyond those we present here, for example, oligodendrocyte transcription factor 2 (71–73).

Our focus here has been on the subpopulation of cells within the tumor that express at least one surface marker associated with the GSC state. Moving forward, mass cytometry antibody panels for GBM that combine assessment of GSCs, other GBM cells, and cells that compose the tumor microenvironment will help refine appropriate targets for therapy (74, 75). For instance, mass cytometry was recently used to characterize leukocyte landscapes in the environments of primary and metastatic brain tumors (76). We envision an integrated approach to diagnostics and therapeutic development that includes assessing single-cell proteomic signaling with RNA and DNA sequencing. By applying these analytics to highly treatment-resistant cells like GSCs, we will better understand the heterogeneous complexity of GBM and how to best target these cells with precision.

## Methods

### Cell lines.

GBM cancer stem cell line 0308 was provided by Howard Fine (Department of Neurology, Weill Cornell Medicine, New York, New York, USA) (37, 77). TS667 from a patient with primary GBM was derived in-house (78), as was MGG8 from a patient with primary GBM (79). 0308 and TS667 cells were cultured in Neurobasal media (Life Technologies) supplemented with 0.5× B27 without vitamin A (Thermo Fisher), 0.5× N2 supplement (Thermo Fischer), 2 mM L-glutamine (Thermo Fisher), 1 mM sodium pyruvate (Thermo Fisher), 50 μg/ml EGF (Peprotec), and 50 μg/ml basic FGF (Peprotec). MGG8 cells were cultured in Neurobasal media (Life Technologies) supplemented with 1× B27 without vitamin A, 1× N2 supplement, 3 mM GlutaMAX (Gibco), 5 mg/ml heparin (Stem Cell Technologies), 20 ng/ml EGF (Peprotec), and 20 μg/ml basic FGF (Peprotec).

### Flow cytometry analysis and clonogenic assay.

10^6^ cells were stained with CD133-APC (4 μl/10^6^ cells, TMP4, Invitrogen), CD44-Alexa Fluor 700 (2 μl/10^6^ cells, BJ18, Biolegend), CD15-FITC (2 μl/10^6^ cells, HI98, Biolegend), and α_6_ integrin-Brilliant Violet 421 (2 μl/10^6^ cells, GoH3, Biolegend) for 15 minutes on ice. Fluorescence-minus-one controls were used. Positive and negative populations were gated according to Supplemental Figure 3. All cell analyses and sorting were performed on a FACS Aria II (BD Biosciences). For the clonogenic assay, we plated 120, 24, 5, and 1 cells per well; 12–18 replicates per dilution in ultralow attachment surface plates. Clonogenic cell frequency was analyzed using ELDA (http://bioinf.wehi.edu.au/software/elda/) (39). GSC subpopulation clonogenic frequencies were analyzed with 1-way ANOVA with Tukey’s post hoc test.

### Cell viability.

Five hundred cells of each GSC subpopulation studied were plated in 96-well plates in triplicates. Cells were treated with increasing concentrations of XAV939 (0.03, 0.1, 0.3, 1, 3, 10, 50 μM; Selleckchem, catalog S1180). Cell viability was measured using CellTiter-Glo (Promega, catalog G7572) after 5 days incubation at 37^o^C. All data were normalized to day 0 and expressed as a relative cell number.

### Patient samples.

Fresh GBM specimens were obtained from freshly resected, excess surgical material from patients at Barnes-Jewish Hospital.

### Tumor dissociation.

Fresh tumor samples were dissociated using Brain Tumor Dissociation Kit (Miltenyi Biotec, catalog 130-095-942) followed by treatment with Myelin Removal Beads II (Miltenyi Biotec, catalog 130-096-733) and Debris Removal Solution (Miltenyi, catalog 130-109-38), according to manufacturer’s instruction. Cells were counted and immediately labeled for mass cytometry analysis.

### Mass cytometry staining and analysis.

3 × 10^6^ cells from GSC lines or from patient samples were stained for mass cytometry as described previously (80) using a panel of 20 antibodies (Table 1) and cisplatin to identify dead cells (81). GSCs were differentiated with 10% FBS in DMEM for 6 weeks as negative controls for cell-surface markers. These cells were run alongside the GSCs. Individual sample read-outs were recorded on a CyTOF2 mass cytometer (Fluidigm). At least 2.5 × 10^5^ events were recorded for each sample and uploaded to Cytobank (http://cytobank.org) (82) for subsequent analysis.

### Mice and tumor implantation.

Human GBM cells (MGG8) were grown in Neurobasal media with supplements as described above. Cells were harvested and dissociated with Accumax (Innovative Cell Technologies) then washed and resuspended in fresh media. GSC subpopulations expressing a single GSC cell-surface marker were enriched using LD columns (Miltenyi Biotec), according to manufacturer’s instruction. For each GSC subpopulation, 30 × 10^6^ cells were incubated with stem cell surface antibody minus the corresponding highly expressed marker. GSCs were incubated on ice for 15 minutes with CD133-APC (4 μl/10^6^ cells, TMP4, Invitrogen), CD44-Alexa Fluor 700 (2 μl/10^6^ cells, BJ18, Biolegend), CD15-Brilliant Violet 605 (2 μl/10^6^ cells, HI98, Biolegend), and α_6_ integrin-Brilliant Violet 421 (2 μl/10^6^ cells, GoH3, Biolegend). After enrichment, GSCs were labeled with the corresponding missing antibody, sorted for the single markers, and immediately implanted.

A total of 500 cells per animal were implanted into 6-week-old NCG female mice (NOD-*Prkdc^em26Cd52^Il2rg^em26Cd22^*/NjuCrl; Charles River Laboratory). Briefly, animals were anesthetized by intraperitoneal injection of ketamine (10 mg/kg) and placed in a stereotactic apparatus (Stoelting). An incision was made over the cranial midline and a burr hole was made 1.5 mm anterior to the lambda and 2.5 mm right of the midline. A 29.5-gauge Hamilton syringe was inserted to a depth of 3 mm and withdrawn 0.5 mm to a depth of 2.5 mm. MGG8 cells (3 μl) were injected over the course of 5 minutes. The incision site was closed by Vetbond (3M).

### Animal monitoring.

Mice were monitored for status daily and sacrificed when neurological deficits became significant.

### Statistics.

All grouped data are presented as mean ± SEM as indicated. All statistical analyses were performed using R version 3.5.0 (R Foundation for Statistical Computing) and the tidyverse library (R package version 1.2.1). Supplemental analysis was performed using Prism 7.0 software (GraphPad). ANOVA with Tukey’s post hoc test was used to assess the significance of differences between each GSC subpopulation in clonogenic assay. Kruskal-Wallis with Mann-Whitney post hoc test was used to assess the significance of non-phospho-β-catenin and pP65 between GSCs and cells with low expression of surface markers. Kruskal-Wallis with Bonferroni’s post hoc tests were used to assess the significance of differences between GSCs grouped by the number of highly expressed surface markers of non–phospho-β-catenin. For animal survival analysis, Kaplan-Meier curves were generated, and log-rank (Mantel-Cox) test was performed to assess difference relative to quadruple-high cells. A *P* value of less than 0.05 was considered significant.

### Study approval.

Approval for the use of human subject material after informed consent was granted by the Institutional Review Board of Washington University School of Medicine in accordance with IRB protocol 201111001. Animal studies were performed in accordance with the recommendations in the *Guide for the Care and Use of Laboratory Animals* (National Academies Press, 2011). The protocols were approved by the Institutional Animal Care and Use Committee at the Washington University School of Medicine (assurance no. A338101). Inoculations were performed under anesthesia induced and maintained with ketamine hydrochloride and xylazine, and all efforts were made to minimize animal suffering.

## Author contributions

LG, AJ, OM, and PD performed the experiments. LG, YEC, AS, KMN, AHK, STO, and MGC analyzed data. JLC, CWB, HW, DDM, and AHK provided key reagents and specimens. LG and MGC wrote the initial draft of the manuscript with the other authors contributing to editing the manuscript into its final form.

## Supplementary Material

Supplemental data

## Figures and Tables

**Table 1:**
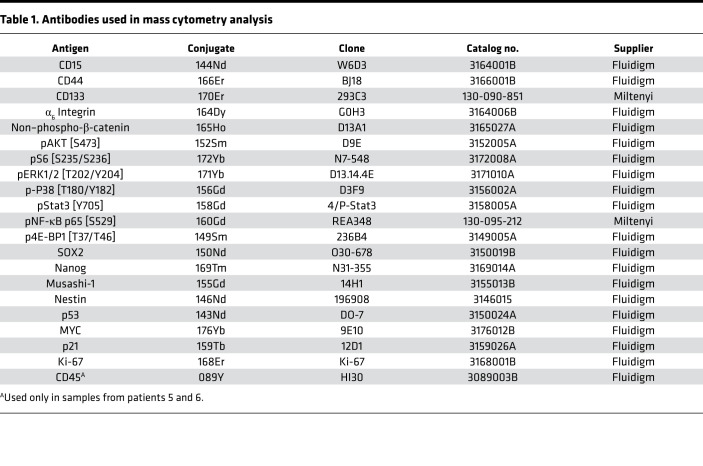
Antibodies used in mass cytometry analysis

**Table 2:**
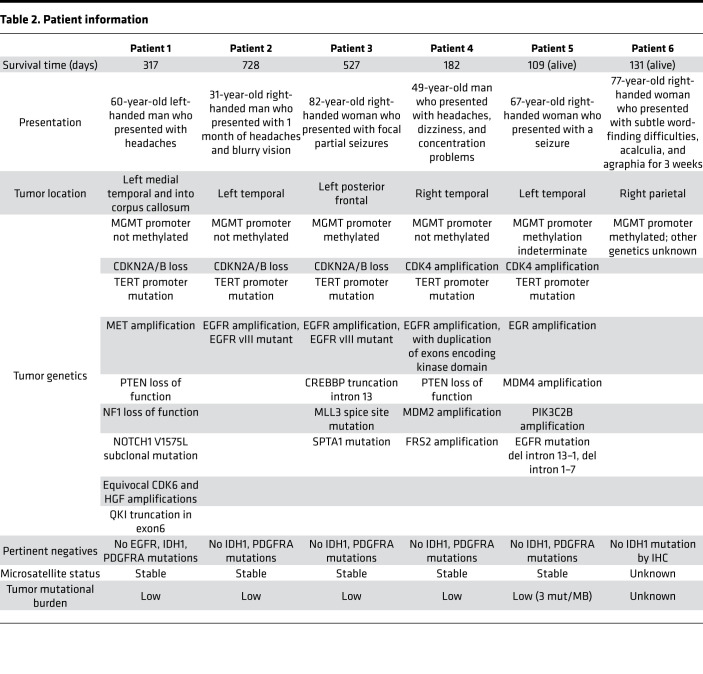
Patient information

**Table 3:**
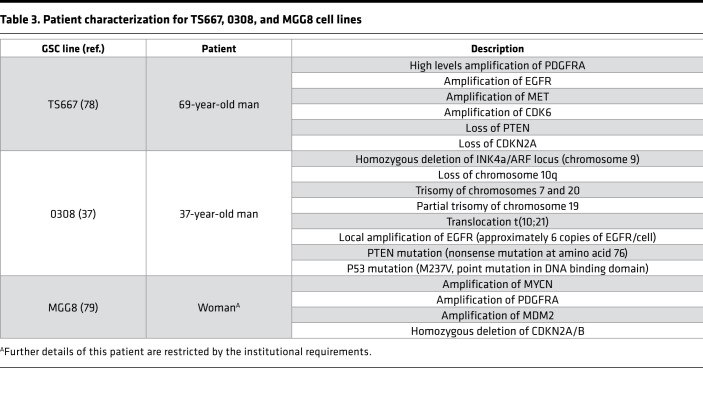
Patient characterization for TS667, 0308, and MGG8 cell lines

## Version 1 01/05/2021

In-Press Preview

## Version 2 02/22/2021

Electronic publication
