# Supplementary material for: Defining phenotypic and functional heterogeneity of glioblastoma stem cells by mass cytometry
Source: JCI Insight. 2021 Feb 22;6(4):e128456. doi: 10.1172/jci.insight.128456 (PMC7934942; doi:10.1172/jci.insight.128456)
Supplement: Supplemental data [file jciinsight-6-128456-s182.pdf]

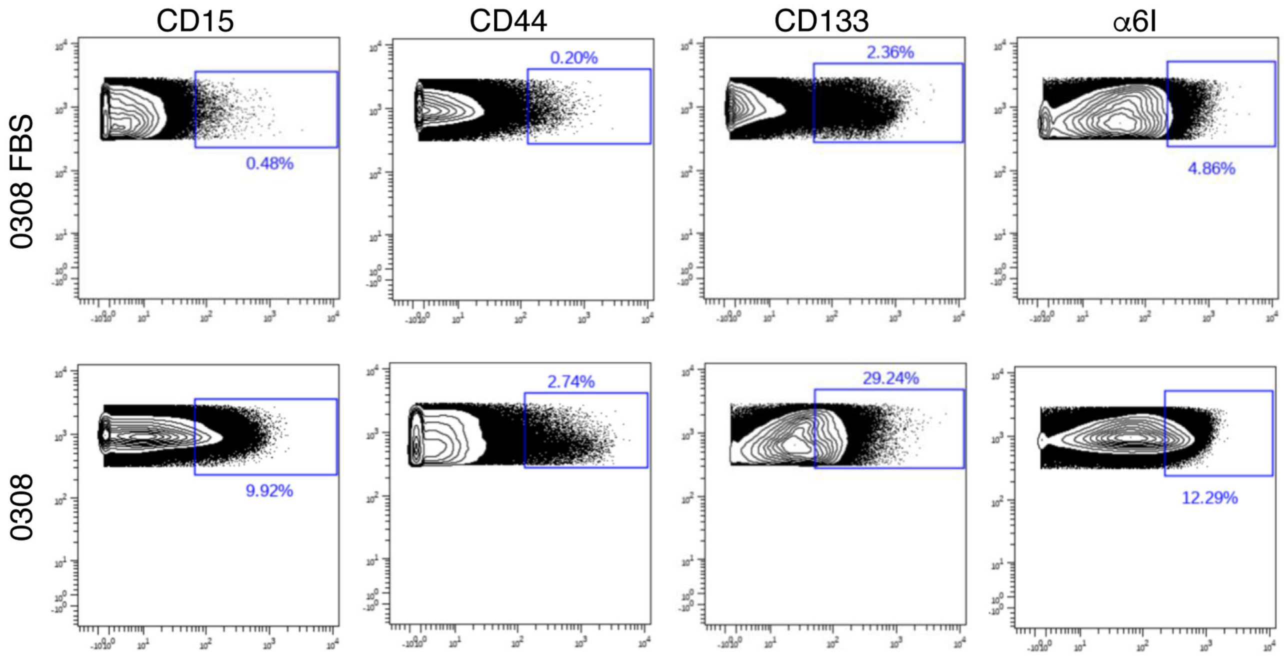

Supplementary Figure 1. Mass cytometry gating strategy for surface marker expression. 0308 GSC line grown in native media (0308) or in the presence of serum (0308 FBS, differentiating condition).

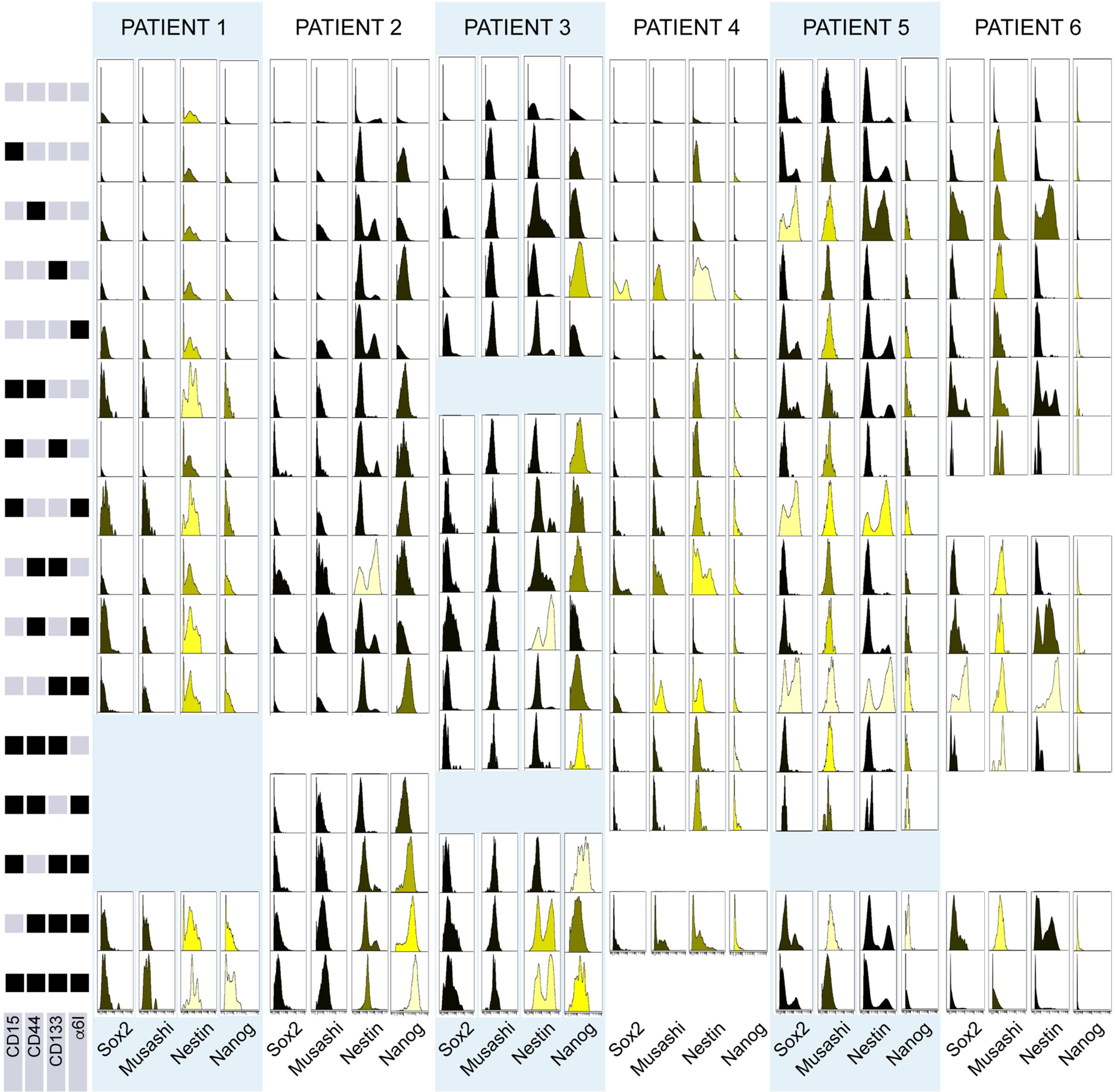

Supplementary Figure 2. Intracellular neural stem cell-associated proteins are expressed in GSC subpopulations and non-GSCs. Histograms indicate protein expression of four intracellular neural stem cell markers (Sox2, Musashi-1, Nestin, and Nanog) in GSC subpopulations from six different patient samples. Left panels show the levels (high, black; low, grey) of the GSC-associated surface markers for each subpopulation.

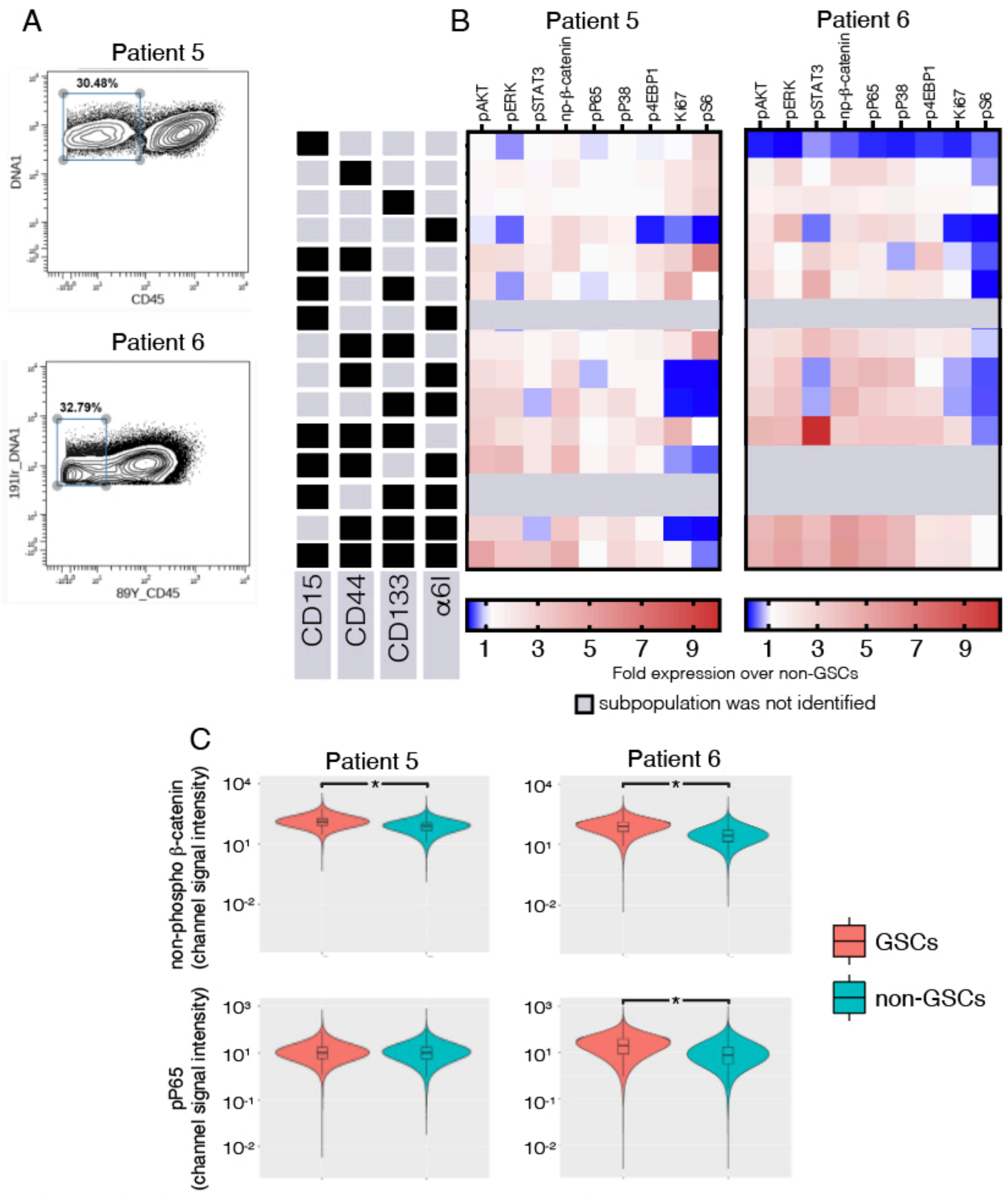

Supplementary Figure 3. The patterns of intracellular signaling remain when CD45^{high} cells are removed. CD45 expression was assessed for samples from patients 5 and 6. (**A**) CD45 gating. (**B**) CD45^{low}CD15^{high}CD44^{high}CD133^{high}a-6integrin^{high} cells have increased activation of ERK and WNT pathways compared to most other GSC subpopulations (**C**) GSCs as a group have higher abundance of non-phospho-b-catenin and phospho-P65 than non-GSCs, after CD45^{high} cells are removed. Kruskall-Wallis with Bonferroni post-hoc test was used; *P<0.05.

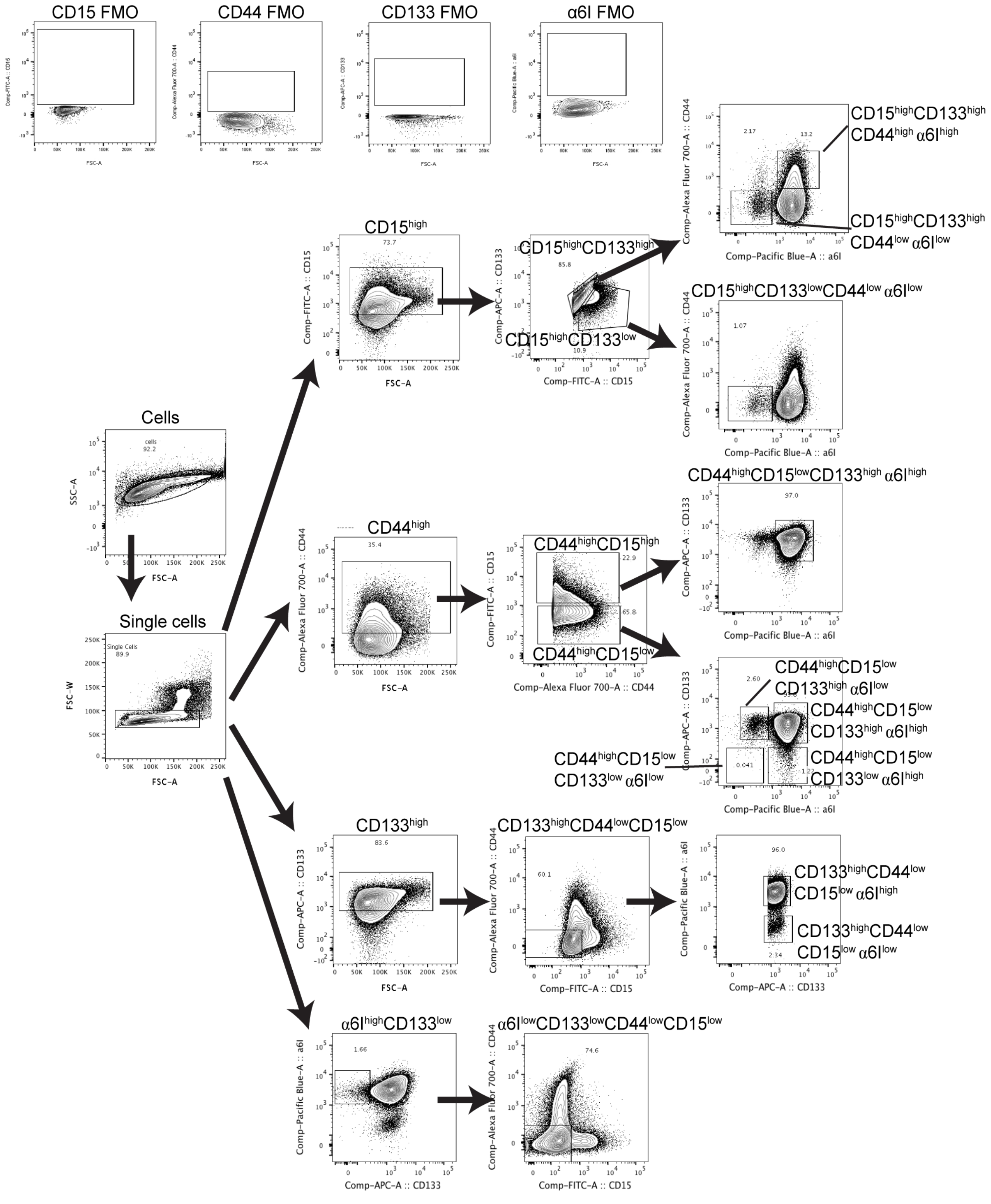

Supplementary Figure 4. Gating strategy for sorting GSC subpopulations by cell surface markers. Top panels indicate fluorescence minus one (FMO) controls used to determine the intensity of positive cells.

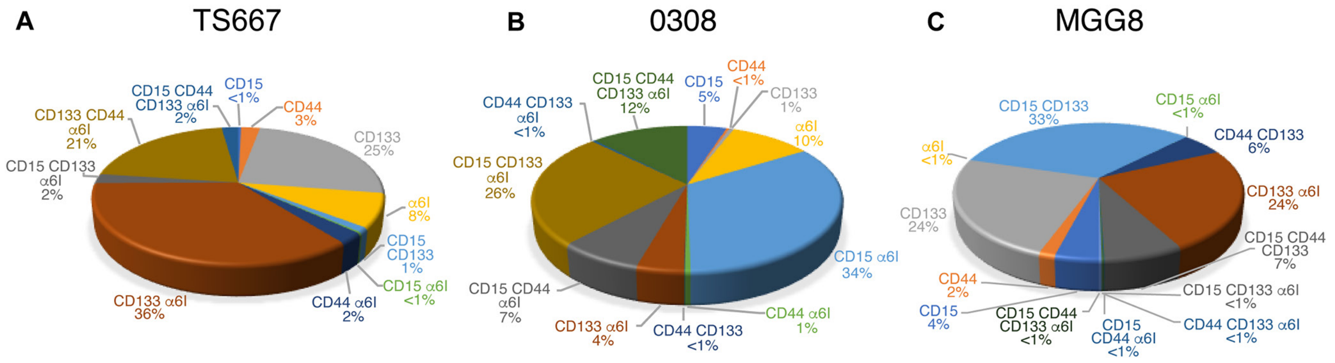

Supplementary Figure 5. Thirteen GSC subpopulations were detected from cells in long-term stem cell media conditions. Pie charts indicate the percentage of each GSC subpopulation relative to the total number of GSCs in (**A**) TS667, (**B**) 0308, (C) MGG8 patient-derived GSC lines.
